# Polymorphisms in Chemokine Receptor 5 and Toll-Like Receptor 3 Genes Are Risk Factors for Clinical Tick-Borne Encephalitis in the Lithuanian Population

**DOI:** 10.1371/journal.pone.0106798

**Published:** 2014-09-16

**Authors:** Auksė Mickienė, Jolita Pakalnienė, Johan Nordgren, Beatrice Carlsson, Marie Hagbom, Lennart Svensson, Lars Lindquist

**Affiliations:** 1 Department of Infectious Diseases, Lithuanian University of Health Sciences, Kaunas, Lithuania; 2 Unit for Infectious Diseases, Department of Medicine, Karolinska Institutet, Karolinska University Hospital Huddinge, Stockholm, Sweden; 3 Division of Molecular Virology, Medical Faculty, Linköping University, Linköping, Sweden; 4 Department of Clinical Microbiology, Linköping University Hospital, Linköping, Sweden; University of Minnesota, United States of America

## Abstract

**Background:**

Tick-borne encephalitis virus (TBEV) infections can be asymptomatic or cause moderate to severe injuries of the nervous system. We previously reported that a nonfunctional chemokine receptor 5 (CCR5) and a functional Toll-like receptor 3 (TLR3) predispose adults to clinical tick-borne encephalitis (TBE). This study expands our previous findings and further examines polymorphisms in *CCR5* and *TLR3* genes in different age and disease severity groups.

**Methods:**

117 children and 129 adults, stratified into mild, moderate and severe forms of TBE, and 103 adults with severe TBE were analyzed. 135 healthy individuals and 79 patients with aseptic meningoencephalitis served as controls. *CCR5 delta 32* and rs3775291 *TLR3* genotypes were established by pyrosequencing, and their frequencies were analyzed using recessive genetic, genotype and allelic models.

**Findings:**

The prevalence of *CCR5Δ32* homozygotes was higher in children (2.5%), in adults with severe TBE (1.9%), and in the combined cohort of TBE patients (2.3%) than in controls (0%) (p<0.05). The nonfunctional homozygous *TLR3* genotype was less prevalent among the combined TBE cohort (11.5%) than among controls (19.9%) (p = 0.025), but did not differ between children TBE and controls. The genotype and allele prevalence of *CCR5* and *TLR3* did not differ in children nor adult TBE cohorts stratified by disease severity. However, in the severe adult TBE cohort, homozygous functional *TLR3* genotype and wt allele were less prevalent compared to the adult cohort with the whole disease severity spectrum (44.4% vs 59.8% p = 0.022 and 65.2% vs 76.4% p = 0.009; respectively).

**Conclusions:**

Independently of age, nonfunctional *CCR5Δ32* mutation is a significant risk factor for development of clinical TBE, but not for disease severity. The polymorphism of *TLR3* gene predisposes to clinical TBE in adults only and may be associated with disease severity. Further studies are needed to clarify the role of these polymorphisms in susceptibility to TBEV infection.

## Introduction

Tick-borne encephalitis (TBE) is a zoonotic disease caused by a RNA virus of the genus *Flavivirus* within the family *Flaviviridae*. Like Japanese encephalitis virus and West Nile virus (WNV), TBE virus (TBEV) is one of the major neurotropic flaviviruses [1]. The endemic area of TBEV ranges from Western Europe to China and Japan, and the virus is taxonomically classified into three subtypes: European, Siberian, and Far Eastern. Members of the European subtype are transmitted primarily by *Ixodes ricinus* ticks and members of the latter two subtypes by *I. persulcatus* [2], [3].

After an infected tick bites a person, TBE viruses first multiply locally in the skin dendritic cells and subsequently are transported to regional nymph nodes. After replication in the lymph nodes, TBE viruses pass into the blood stream. Various organs are invaded after a hematogenic spread of the viruses, and the release of TBEV from various tissues enables the viremia to continue for several days. Although in the majority of cases the TBEV infection is terminated at the extraneural stage, in some cases high virus replication in the primarily affected organs leads to a progressive infection when TBEV crosses the blood-brain barrier and invades the brain [4].

The clinical spectrum of TBEV infection varies considerably from asymptomatic to mild meningitis, severe encephalitis or encephalomyelitis [5]. Why certain individuals respond with severe clinical symptoms while the majority either remains asymptomatic or only develops mild disease is largely unknown.

Recently, we have shown an association between development of TBE and the polymorphisms of two genes modulating the innate immune response. The first genetic risk factor identified for TBE was a 32-bp deletion in the coding region of the chemokine receptor 5 (*CCR5*) gene [6]. The *CCR5* gene encodes a protein expressed on the surfaces of leukocytes that is involved in regulation of leukocyte migration [7], [8]. The second risk factor was a rs3775291 single nucleotide polymorphism (SNP) Leu412Phe in exon 4 of the Toll-like receptor 3 (*TLR3*) gene [9]. TLR3 protein is expressed in dendritic cells, a variety of epithelial cells, and in the brain, and is localized primarily in endosomal membranes [10]. The TLR3 protein binds to virus-derived dsRNA and activates the transcription factors, NF-κB and IRF3. This in turn induces the production of type 1 interferons and pro-inflammatory cytokines, in particular TNF-α, which decreases the integrity of the blood-brain barrier and allows the passage of the virus into the brain [10]–[12].

The aim of this study was to extend our previous findings by enlarging our data set, to understand the role of these polymorphisms in different age groups, and to investigate if they determine the severity of TBE.

## Materials and Methods

### Study population

#### TBE cases

All patients included in the study had clinical signs of neuroinfection and pleocytosis in cerebrospinal fluid (CSF) ≥8×10^6^/l. TBE was diagnosed by the demonstration of specific IgM activity by the two-step ELISA method in serum samples [13]. The clinical presentation of TBE was classified as mild, moderate or severe, as previously described [14]. Briefly, mild form showed predominantly meningeal symptoms; moderate form showed monofocal encephalitic symptoms (ataxia, dysphasia, tremor, single cranial nerve paralysis) and/or moderate diffuse brain dysfunction (Glasgow Coma Scale (GCS) score >9); severe form showed multifocal encephalitic symptoms and/or GCS ≤9. For all study participants, CSF specimens were obtained by lumbar puncture in a standardized manner on a median of 1 day (range, 0–11 days) after admission to the hospital. Immediately after collection, all CSF samples were examined for cell count, differential count, total protein and glucose concentration.

Three TBE cohorts were used in this study.

The first cohort consisted of all consecutive patients under 18 years of age admitted from 1999 through 2009 (n = 117), 73 of which were classified as mild, 40 as moderate, and 4 as severe TBE; and is called “Children TBE cohort” in the manuscript.

The second cohort consisted of all adults meeting the criteria of severe form of disease from the database of 831 consecutive adult TBE patients admitted from 2004 through 2010 (n = 103); and is called “Adult severe TBE cohort” in the manuscript.

The third TBE patient cohort was composed of 129 adult TBE patients, stratified by the same clinical classification into mild (n = 56), moderate (n = 57), and severe (n = 16) form of TBE, and reported earlier in our previous studies [6], [9], [14].

Demographic data of all three TBE cohorts are presented in Table 1.

**Table 1:** Demographic data of children TBE, adult severe TBE, and adult TBE cohorts.

Characteristic	Value	p
Children TBE (n = 117)		
Gender, n (%):		
Boys	70 (59.2)	p = 0.033
Girls	47 (40.2)	
Age, mean (years)±SD:		
Boys	12.07±3.857	p = 0.376
Girls	11.43±3.855	
		
Mild form	11.36±3.987	p = 0.179
Moderate form	12.73±3.289	
Severe form	11.00±5.888	
Adult severe TBE (n = 103)		
Gender, n (%):		
Male	55 (53.4)	p = 0.490
Female	48 (46.6)	
Age, mean (years)±SD:		
Male	51.93±15.419	p = 0.080
Female	57.27±15.108	
Adult TBE (n = 129)		
Gender, n (%):		
Male	70 (54.3)	p = 0.333
Female	59 (45.7)	
Age, mean (years)±SD:		
Male	43.56±15.284	p = 0.176
Female	46.97±12.756	
		
Mild form	41.34±14.895	p = 0.021
Moderate form	47.30±13.450	
Severe form	50.56±11.696	

Three combined cohorts of TBE cases were made for statistical analysis.

The first combined cohort was created in order to cover the entire age and disease severity spectrum in usual proportions of mild, moderate, and severe forms, and consisted of the 117 children recruited in the present study and 129 adults reported previously [6], [9], [14].

The second combined cohort was a total sample of all three TBE cohorts described above.

The third combined cohort was used to investigate the association of gene polymorphisms and severity of TBE, and consisted of an overall cohort of adults with TBE (129 reported previously and 103 severe TBE cases).

#### Controls

Two control cohorts from our previous studies on TBE were included for comparison.

The first control cohort was 135 healthy Lithuanians with no documented TBEV infection matched geographically and by age and gender.

The second control cohort was 79 geographically matched adult patients with aseptic meningoencephalitis (AME), negative for IgM antibody for TBE virus.

As no difference in *CCR5Δ32* and *TLR3* rs3775291 genotype and allele distribution was found between these two control cohorts [6], [9], they were considered as one combined control cohort when statistical calculations were performed.

### *CCR5Δ32* and *TLR3* rs3775291 genotyping

All serum samples were analyzed for a 32-bp deletion in the coding region of the chemokine receptor *CCR5* gene and for missense mutation rs3775291 (G/A, Leu412Phe) in exon 4 of the Toll-like receptor *TLR3* gene by polymerase chain reaction (PCR) and pyrosequencing, essentially as described in a previous articles [6], [9]. DNA was extracted from serum by QiaAmp 96 DNA Blood Kit (Qiagen), in accordance with the manufacturer's instructions and the DNA samples were eluted into 100 µL of AE buffer (Qiagen) and stored at −20°C, until further analyzed.

For PCR, we used 5–7.5 µL DNA, 2.5 µL 10xPCR buffer II (Applied Biosystems), 2.5 µL 50 mM MgCl (Applied Biosystems), 1 uL 10 mM GeneAmp dNTP mix with dTTP (Applied Biosystems), 1(*CCR5*) or 2 (*TLR3*) µL 10 µM Fw, 1 (*CCR5*) or 2 (*TLR3*) µL 10 µM Rev primer (Table 2), and 0.2 µL AmpliTaq Gold DNA Polymerase per reaction. The final volume was adjusted with ultraclean water to a final volume of 25 µL. The PCR reaction was performed at 95°C for 5 min followed by 50 cycles of 15 s at 95°C, 30 s at 55 or 65°C depending on the amplified sequence (Table 2), and 30 s at 72°C, and finally 1 cycle of 5 min at 72°C.

**Table 2:** Primers, annealing temperatures and dispension orders used for amplification and sequencing of the different mutations.

Mutation (gene)	PCR and sequencing primers	PCR annealing temperature	Dispension order
*Δ32 CCR5*		65°C	GACAGTCAGA
	Fw: 5′-CACCTGCAGCTCTCATTTTCC-3′		
	Rev: 5′-BIOTIN- TTTTTAGGATTCCCGAGTAGCA-3′		
	Seq: 5′-CAGCTCTCATTTTCCAT-3′		
rs3775291 *TLR3*		55°C	GAGTATGT
	Fw: 5′-TCATTAAGGCCCAGGTCAAG-3′		
	Rev:5′-BIOTIN-TGGCTAAAATGTTTGGAGCA-3′		
	Seq: 5′- TTATTCTTGGTTAGGTTGA-3′		

PCR – polymerase chain reaction, Fw – forward, Rev – reverse, Seq – sequencing.

Pyrosequencing to distinguish between homozygous wild type (wt/wt), heterozygous (wt/mut), and homozygous mutant (mut/mut) of *CCR5Δ32* and *TLR3* rs3775291 genotypes was performed in a PSQ 96 MA Instrument (Biotage) as described previously [6]. Sequencing primers and dispension orders are shown in Table 2. The *CCR5Δ32* genotype of a subset of DNA samples (n = 30) were verified by gel electrophoresis, by separation of the 100- and 132-bp PCR amplicons in a 2% agarose gel. The *TLR3* genotype for 8 DNA samples could not be determined by pyrosequencing and were determined by sanger sequencing using the same amplicon with forward and reverse primers (Table 2) as sequencing primers.

### Statistical methods

Statistical analysis included descriptive statistics with frequency analysis (percentages) for categorical variables and means with standard deviations (SD) for continuous variables. The two-sample *t*, Mann-Whitney or Kruskal-Wallis tests were used for continuous variables as appropriate. Proportions were compared using *χ*2 or Fisher's exact tests. One way analysis of variance (ANOVA) was used to compare more than two independent groups. Multivariate logistic regression model was used to investigate associations of risk factors with disease severity. Statistical analyses were performed using SPSS (*Statistical Package for the Social Science for Windows; Chicago, USA*) 13.1 software. p<0.05 was considered statistically significant.

For the *CCR5* gene, a recessive genetic model (i.e., *CCR5Δ32* homozygotes vs wild-type *CCR5* plus *CCR5Δ32* heterozygotes) and an allelic model (mut vs wt) were applied. For *TLR3* rs3775291, genotype (3×2 contingency analysis) was used as well as allelic model (mut vs wt). For the cohort of all adult patients stratified by severity of TBE, a recessive genetic model was also applied. Statistical calculations included only samples that were successfully genotyped.

### Ethical approval

The study was approved by Kaunas Regional Research Ethics Committee (No. BE-2-15) and a written informed consent was obtained from each study participant or from a parent/legally authorised representative for all children.

## Results

### *CCR5Δ32* polymorphism in clinical TBE

#### Children TBE cohort

All child patients with TBE (n = 117) were successfully genotyped.

The prevalence of *CCR5Δ32* homozygotes was higher in the children TBE cohort (2.5% (3/117) than in the Lithuanian TBEV-naive control (0% (0/134) and in the AME cohort (0% (0/76). The difference was also observed when the children TBE cohort was compared with the combined control cohort (p = 0.045) (Table 3).

**Table 3:** Genotype distribution of *CCR5* and *Δ32* allele prevalence among TBE patients, Lithuanian TBE virus-naive control subjects, and patients with aseptic meningoencephalitis (AME) of non-TBEV etiology.

Cohort	Population	*CCR5* genotype, n (%)			Failed, n (%)	Allele prevalence (wt/Δ32 allele)	Reference
		wt/wt	wt/Δ32	Δ32/Δ32			
TBE1	Children TBE (n = 117)	91 (77.8)	23 (19.7)	3 (2.5)^a^	0 (0)	0.876/0.124	
TBE2	Adult severe TBE (n = 103)	83 (80.6)	18 (17.5)	2 (1.9)^b^	0 (0)	0.890/0.110	
TBE3	Adult TBE (n = 129)	97 (75.2)	29 (22.5)	3 (2.3)	0 (0)	0.864/0.136	[6]
TBE1+TBE3	Children and adult TBE (n = 246)	188 (78.4)	52 (21.1)	6 (2.4)^c^	0 (0)	0.870/0.130^f^^,^ g	
TBE1+TBE2+TBE3	All TBE cases (n = 349)	271 (77.7)	70 (20.1)	8 (2.3)^d^	0 (0)	0.877/0.123^h^^,^ i	
C1	Lithuanian controls (n = 134)	112 (83.6)	22 (16.4)	0 (0)	0 (0)	0.918/0.082^f^^,^ h	[6]
C2	Adult AME (n = 79)	60 (78.9)	16 (21.1)	0 (0)	3 (3.8)	0.895/0.105	[6]
C1+C2	Lithuanian controls and adult AME (n = 213)	172 (81.9)	38 (18.1)	0 (0)^a^^,^ b ^,^ c ^,^ d	3 (1.4)	0.910/0.090^g^^,^ i	[6]

wt – wild type.

a TBE1 vs C1+C2: p = 0.045 (Pearson's χ^2^ test).

b TBE2 vs C1+C2: p = 0.043 (Pearson's χ^2^ test).

c TBE1+TBE3 vs C1+C2: p = 0.023 (Pearson's χ^2^ test).

d TBE1+TBE2+TBE3 vs C1+C2: p = 0.027 (Pearson's χ^2^ test).

f TBE1+TBE3 vs C1: p = 0.046 (Pearson's χ^2^ test) and OR = 1.672 (95% CI 1.005–2.782; p = 0.048).

g TBE1+TBE3 vs C1+C2: p = 0.059 (Pearson's χ^2^ test).

h TBE1+TBE2+TBE3 vs C1: p = 0.069 (Pearson's χ^2^ test).

i TBE1+TBE2+TBE3 vs C1+C2: p = 0.091 (Pearson's χ^2^ test).

The *CCR5Δ32* allele prevalence was also higher in the children TBE cohort (12.4% (29/234) than in the Lithuanian TBEV-naive control (8.2% (22/268) and in the AME cohort (10.5% (16/152) (Table 3) but the difference was not statistically significant when the children TBE cohort was compared with the control cohorts.

#### Adult severe TBE cohort

All adult patients with severe TBE (n = 103) were successfully genotyped.

In this cohort, 2 *CCR5Δ32* homozygotes were found, making the prevalence of *CCR5Δ32* homozygotes higher in the TBE cohort (1.9% (2/103) than in the Lithuanian TBEV-naive control and in the AME cohorts (Table 3). Also, the prevalence of *CCR5Δ32* homozygotes differed when the TBE cohort was compared with the combined control cohort (p = 0.043) (Table 3).

The *CCR5Δ32* allele prevalence was also higher in the cohort of adult patients with severe TBE (11% (22/206) than in the Lithuanian TBEV-naive control and in the AME cohorts (Table 3), but no statistical differences were found between the TBE cohort compared to the control cohorts.

#### Combined TBE cohorts

Altogether, 8 *CCR5Δ32* homozygotes were found among the combined cohort of all patients with TBE (2.3% (8/349)). The prevalence of *CCR5Δ32* homozygotes was higher in this cohort than among the AME and the Lithuanian TBE-naive control cohorts (Table 3). The difference was statistically significant when both the cohort of all TBE patients (n = 349) and the combined cohort of children and adult TBE cases (n = 246) were compared with the combined control cohort (n = 210; p = 0.027 and p = 0.023, respectively) (Table 3).

The *CCR5Δ32* allele prevalence was also higher in the cohort of all patients with TBE (n = 349) (12.3% (86/698)) than in the AME cohort, and the Lithuanian TBE-naive control cohort (Table 3). The difference was observed when the combined children and adult TBE cohort (n = 246) was compared with the cohort of Lithuanian TBE-naive individuals (p = 0.046), which suggests *CCR5Δ32* allele being a risk factor for clinical TBEV infection (Odds ratio (OR) 1.672; 95% confidence interval (CI) 1.005–2.782; p = 0.048) (Table 3). The same trend was observed when the cohort of all TBE cases (n = 349) was compared with the Lithuanian TBE-naive cohort (p = 0.069) as well as in comparison of the combined children and adult TBE cohort (n = 246) with the combined control cohort (p = 0.059) (Table 3).

### *TLR3* polymorphism in clinical TBE

#### Children TBE cohort

105 out of 117 (89.7%) children with TBE were successfully genotyped.

For *TLR3* rs3775291, the genotype distribution in children cohort was 51.4% for homozygous wild type, 34.3% for heterozygous, 14.3% for mutant homozygous genotype, and was in concordance with the distribution in the cohort of the Lithuanian TBE-naive controls (51.6% wt/wt, 29.4% heterozygous, 19.0% mut/mut, respectively), as well as in the AME cohort (46.7% wt/wt, 32.0% heterozygous, 21.3% mut/mut, respectively). When the children TBE cohort was compared with the combined control cohort, we did not find any significant differences (p = 0.453). The wt allele prevalence between the children TBE and the control cohorts did not differ either (Table 4).

**Table 4:** Genotype distribution of *TLR3* rs3775291 and allele prevalence among TBE patients, Lithuanian TBEV-naive control subjects, and patients with aseptic meningoencephalitis (AME) of non-TBEV etiology.

Cohort	Population	*TLR3* rs3775291 genotype, n (%)			Failed, n (%)	Allele prevalence (wt/mut allele)	Reference
		wt/wt	wt/mut	mut/mut			
TBE1	Children TBE (n = 117)	54 (51.4)	36 (34.3)	15 (14.3)^a^	12 (10.3)	0.686/0.314	
TBE2	Adult severe TBE (n = 103)	44 (44.4)^f^	41 (41.4)	14 (14.1)^b^	4 (3.9)	0.652/0.348^g^	
TBE3	Adult TBE (n = 128)	76 (59.8)^f^	42 (33.1)	9 (7.1)	1 (1.0)	0.764/0.236^g^	[9]
TBE1+TBE3	Children and adult TBE (n = 245)	130 (56.0)	78 (33.6)	24 (10.4)^c^	13 (5.3)	0.728/0.272^e^	
TBE1+TBE2+TBE3	All TBE cases (n = 348)	174 (52.5)	119 (36.0)	38 (11.5)^d^	17 (4.8)	0.705/0.295	
C1	Lithuanian controls (n = 135)	65 (51.6)	37 (29.4)	24 (19.0)	9 (7.0)	0.663/0.337	[9]
C2	Adult AME (n = 77)	35 (46.7)	24 (32.0)	16 (21.3)	2 (3.0)	0.627/0.373	[9]
C1+C2	Lithuanian controls and adult AME (n = 212)	100 (49.8)	61 (30.3)	40 (19.9)^a^^,^ b ^,^ c ^,^ d	11 (5.2)	0.649/0.351^e^	[9]

wt – wild type.

a TBE1 vs C1+C2: p = 0.453 (Pearson's χ^2^ test).

b TBE2 vs C1+C2: p = 0.135 (Pearson's χ^2^ test).

c TBE1+TBE3 vs C1+C2: p = 0.02 (Pearson's χ^2^ test).

d TBE1+TBE2+TBE3 vs C1+C2: p = 0.025 (Pearson's χ^2^ test).

e TBE1+TBE3 vs C1+C2: OR = 1.449 (95% CI 1.085–1.936; p = 0.012).

f TBE2 vs TBE3: p = 0.022 (Pearson's χ^2^ test).

g TBE2 vs TBE3: p = 0.009 (Pearson's χ^2^ test).

#### Adult severe TBE cohort

*TLR3* rs3775291 genotypes were successfully obtained for 99 out of the cohort of 103 (96.1%) adults with severe TBE.

In this TBE cohort, the genotype distribution of *TLR3* rs3775291 was 44.4% for homozygous wild type, 41.4% for heterozygous, 14.1% for mutant homozygous genotype, and was in concordance with the distribution among the cohort of Lithuanian TBE-naive controls and among the AME cohort, and did not differ when compared with the combined control cohort (p = 0.135). The wt allele distribution between the TBE and the control cohorts did not differ either (Table 4).

#### Combined TBE cohorts

The mutant homozygous genotype for TLR3 rs3775291 was found significantly less frequently among TBE patients in both the combined cohort of children and adults (n = 232) and the overall combined cohort of TBE cases (n = 331), compared to the combined control cohort (p = 0.02 and p = 0.025, respectively) (Table 4). The wild allele was found to be a risk factor for clinical TBEV infection when comparing the children and adult TBE cohort (n = 232) with the combined control cohort (OR 1.449, 95% CI 1.085–1.936, p = 0.012).

### *CCR5* and *TLR3* polymorphisms and severity of TBE

An association between *CCR5* and *TLR3* polymorphisms and the severity of TBE was analysed in the children cohort and the overall cohort of adults, stratified by severity of disease.

We did not detect any significant difference in *CCR5* genotype distribution and in *CCR5Δ32* allele prevalence among the cohort of children (n = 117) and adults (n = 232), stratified by severity of disease (Table 5).

**Table 5:** Genotype distribution of *CCR5* and *Δ32* allele prevalence among patients with TBE, stratified by severity of disease.

Population	Clinical form of TBE	*CCR5* genotype, n (%)			Allele prevalence (wt/Δ32 allele)
		wt/wt	wt/Δ32	Δ32/Δ32	
Children TBE*	Mild (n = 73)	58 (79.5)	14 (19.2)	1 (1.3)	0.890/0.110
	Moderate (n = 40)	30 (75.0)	8 (20.0)	2 (5.0)	0.850/0.150
	Severe (n = 4)	3 (75.0)	1 (25.0)	0 (0)	0.875/0.125
	Total (n = 117)	91 (77.8)	23 (19.7)	3 (2.5)	0.876/0.124
Adult TBE**	Mild (n = 56)	43 (76.8)	13 (23.2)	0 (0)	0.884/0.116
	Moderate (n = 57)	44 (77.2)	10 (17.5)	3 (5.3)	0.860/0.140
	Severe (n = 119)	93 (78.1)	24 (20.2)	2 (1.7)	0.882/0.118
	Total (n = 232)	180 (77.6)	47 (20.2)	5 (2.2)	0.877/0.123

wt – wild type; M – Mild, Mo – Moderate, S – Severe form.

* (wt/wt + wt/Δ32) vs Δ32/Δ32, M vs Mo vs S: p = 0.479; M vs (Mo+S): p = 0.292; wt vs Δ32, M vs Mo vs S: p = 0.571; M vs (Mo+S): p = 0.391.

** (wt/wt + wt/Δ32) vs Δ32/Δ32, M vs Mo vs S: p = 0.137; M vs (Mo+S): p = 0.202; wt vs Δ32, M vs Mo vs S: p = 0.806; M vs (Mo+S): p = 0.802.

For *TLR3* rs3775291, neither the genotype distribution nor the wild type allele prevalence differed in children (n = 105) and adult (n = 226) TBE cohorts (Table 6). Interestingly, the cohort of adults with severe TBE (n = 99) had a significantly lesser prevalence of both homozygous wild genotype and wt allele compared with the cohort of adult TBE cases (n = 127), (44.4% vs 59.8% p = 0.022 and 65.2% vs 76.4% p = 0.009; respectively) (Table 4). Furthermore, the prevalence of homozygous mutation was higher in adults with severe or moderate TBE compared to adults with mild TBE (p = 0.071, Table 6).

**Table 6:** Genotype distribution of *TLR3* rs3775291 and allele prevalence among patients with TBE, stratified by severity of disease.

Population	Clinical form of TBE	*TLR3* rs3775291 genotype, n (%)			Allele prevalence (wt/mut allele)
		wt/wt	wt/mut	mut/mut	
Children TBE*	Mild (n = 66/73)	31 (46.9)	25 (37.8)	10 (15.2)	0.659/0.341
	Moderate (n = 36/40)	21 (58.3)	10 (27.8)	5 (13.9)	0.722/0.278
	Severe (n = 3/4)	2 (66.7)	1 (33.3)	0 (0)	0.833/0.167
	Total (n = 105/117)	54 (51.4)	36 (34.3)	15 (14.3)	0.686/0.314
Adult TBE**	Mild (n = 54/56)	32 (59.3)	20 (37.0)	2 (3.7)	0.778/0.222
	Moderate (n = 57/57)	33 (57.9)	17 (29.8)	7 (12.3)	0.728/0.272
	Severe (n = 115/119)	55 (47.8)	46 (40.0)	14 (12.2)	0.678/0.322
	Total (n = 226/232)	120 (53.1)	83 (36.7)	23 (10.2)	0.715/0.285

wt – wild type; M – Mild, Mo – Moderate, S – Severe form.

* wt/wt vs wt/mut vs mut/mut, M vs Mo vs S: p = 0.757; M vs (Mo+S): p = 0.485; wt vs mut, M vs Mo vs S: p = 0.476; M vs (Mo+S): p = 0.280.

** wt/wt vs wt/mut vs mut/mut, M vs Mo vs S: p = 0.264; M vs (Mo+S): p = 0.180; (wt/wt + wt/mut) vs mut/mut, M vs Mo vs S: p = 0.197; M vs (Mo+S): p = 0.071; wt vs mut, M vs Mo vs S: p = 0.157; M vs (Mo+S): p = 0.096.

### Correlation between demographic, laboratory, and genetic parameters and severity of TBE

CSF leukocyte and mononuclear cell count and total protein level in TBE patient cohorts stratified by severity of disease (excluding CSF samples with red blood cells due to traumatic lumbar puncture) are presented in Table 7.

**Table 7:** CSF leukocyte and mononuclear cell count and total protein level in TBE patient cohorts, stratified by severity of disease.

Population	Clinical form, (n)	Leukocyte count (10^6^/l), mean±SD (min–max)	Mononuclear cells (10^6^/l), mean±SD (min–max)	Total protein (g/l), mean±SD (min–max)
Children TBE (n = 117)*		182.06±149.14 (8–853)	111.15±87.85 (1–428)	0.585±0.309 (0.137–1.630)
	Mild (n = 73)	167.71±132.53 (8–612)	104.81± 86.96 (5–428)	0.569±0.307 (0.137–1.630)
	Moderate (n = 40)	217.20±176.53 (10–853)	128.51±90.69 (1–367)	0.630±0.324 (0.230–1.620)
	Severe (n = 4)	92.50±33.79 (64–141)	53.37±24.05 (19–71)	0.430±0.178 (0.190–0.580)
Adult severe TBE (n = 89/103)		180.35±218.14 (8–1600)	106.09±88.74 (7–418)	0.892±0.409 (0.214–2.540)
Adult TBE (n = 117/129)**		154.49±163.77 (9–995)	113.79±94.59 (9–512)	0.688±0.319 (0.237–1.908)
	Mild (n = 52)	151.92±170.27 (9–995)	118.30±105.13 (9–428)	0.688±0.282 (0.241–1.543)
	Moderate (n = 52)	143.44±145.17 (10–682)	106.05±90.45 (10–512)	0.652±0.324 (0.237–1.526)
	Severe (n = 13)	208.92±206.71 (30–832)	123.98±69.87 (19–245)	0.833±0.424 (0.388–1.908)
All TBE cases (n = 323/349)***		171.60±175.63 (8–1600)	110.57±90.16 (1–512)	0.707±0.364 (0.137–2.540)
	Mild (n = 125)	161.14±148.94 (8–995)	110.02±94.19 (5–428)	0.618±0.301 (0.137–1.630)
	Moderate (n = 92)	175.51±162.85 (10–853)	116.75±90.72 (1–512)	0.643±0.322 (0.230–1.620)
	Severe (n = 106)	180.54±212.58 (8–1600)	106.29±85.55 (7–418)	0.868±0.412 (0.190–2.540)

Note. Normal CSF cell count range: 0–5×10^6^/l leukocytes (all mononuclear cells), no red blood cells; normal total protein range: 0.15–0.45 g/l.

M – Mild, Mo – Moderate, S – Severe form.

* M vs Mo vs S, Leukocyte count: p = 0.114; Mononuclear cells: p = 0.160; Total protein: p = 0.362.

** M vs Mo vs S, Leukocyte count: p = 0.434; Mononuclear cells: p = 0.763; Total protein: p = 0.191.

*** M vs Mo vs S, Leukocyte count: p = 0.684; Mononuclear cells: p = 0.729; Total protein: p<0.001.

In the overall combined cohort of TBE cases (n = 349), CSF cell count, homozygous *CCR5Δ32* genotype, Δ32 allele, homozygous wild type, homozygous mutant *TLR3* rs3775291 genotype and wt allele did not correlate with the severity of TBE.

Three independent predictors of the encephalitic (moderate and severe) form of illness in this cohort – age, gender and total protein in CSF – were assessed using the multivariate logistic regression model. Increased age (with each year added, OR = 1.045; 95% CI 1.031–1.059, p<0.001), increased total protein in CSF (with each g/l added, OR = 2.353; 95% CI 1.039–5.328, p = 0.04) and being female (OR = 1.714; 95% CI 1.019–2.880, p = 0.042) were associated with the risk for encephalitic (moderate and severe) form of TBE.

## Discussion

This study both confirms and extends our previous findings that a nonfunctional CCR5 protein and a functional TLR3 receptor are associated with the clinical expression of TBE. The nonfunctional CCR5 protein predisposes to the clinical TBE independently of age but does not determine the severity of TBE. In contrast to *CCR5*, the polymorphism of *TLR3* gene plays a role in the development of clinical TBE in adults only and could also be associated with disease severity.

The rationale to extend our previous studies was based on the hypothesis that genetic factors predispose to the important features of TBE: the disease being much more common in adults than in children [5], [15], [16], and having a very broad spectrum of disease severity. The study was designed to avoid selection bias combining two large prospective and consecutive cohorts of children and adults with severe TBE using the same clinical classification by the same investigators.

In this report, the frequency of *CCR5*Δ*32* homozygotes was higher in TBE patients than in controls (2.5% in children, 1.9% in adults with severe TBE, and 0% in controls), as in agreement with the results of our first study on the association between *CCR5Δ32* and TBE [6]. Furthermore, demonstrating this association in the large combined children and adult TBE cohort, covering the whole age and disease severity spectrum, strongly suggests that functional CCR5 protein plays a role in the host defense against TBE infection. The association between functional/wild type *TLR3* polymorphism and TBE in the combined cohorts was consistent with the previous observations. However, in children and in adults with severe TBE, the homozygous wild *TLR3* genotype was not more prevalent than in the controls. *TLR3* polymorphism in TBE in children has never been investigated before, and our results indicate that, in contrast to adults, this particular gene polymorphism is not a risk factor predisposing to clinical TBE in children.

In addition to genotype-disease association, the importance of the mutated allele has also been shown in our study. As the prevalence of both *CCR5Δ32* and wild type *TLR3* alleles were higher among TBE patients than the controls, we believe that a gene-dosage effect of those proteins exists in TBEV infection, and that heterozygote carriers are predisposed to clinical TBE as well. Our findings on *CCR5Δ32* contradict those of clinical studies on WNV infection which claim that both *CCR5Δ32* alleles are needed to confer a deficit [17], [18], but are in agreement with the data on HIV that show slower rates of disease progression to AIDS in heterozygous *CCR5Δ32* individuals [19]. Data on TLR3, and on functional SNP, characterized by amino acid substitution from phenylalanine to leucine at position 412 in particular, are also inconsistent and depend on the virus under consideration. Gorbea et al. could only prove the wild type homozygosity to be significantly associated with enteroviral myocarditis [20]. However, a recent study on chronic hepatitis C in liver transplant patients demonstrated that heterozygous carriers of the mutated allele are predisposed to higher mortality after liver transplantation [21]. Also, heterozygous variant of the same SNP in *TLR3* gene was associated with low antibody and lymphoproliferative responses to measles vaccination [22] and with early mortality and an accelerated decline in lung function in idiopathic pulmonary fibrosis patients [23].

To the best of our knowledge, only one other research group has studied the role of human genetics in TBE [24]–[26]. In the recently published study, no association between *CCR5* polymorphism and predisposition to TBE was found in the Russian population [26]. Although the findings on TLR3 in the Russian report were in agreement with our data, major differences in design make comparison of the results difficult.

A special focus of this study was gene polymorphisms predisposing to disease severity. No evidence of such association for *CCR5Δ32* in neither of our cohorts was found. In contrast, a recent study on WNV established *CCR5Δ32* homozygosity as a predictor of severity of clinical presentation [27]. Although being large, our study may still have been inadequate in terms of the number of *CCR5Δ32* homozygotes to demonstrate this association. An unexpected finding of our study was that both homozygous wild *TLR3* genotype and wt allele were significantly less prevalent in the cohort of adults with severe form of TBE compared to the cohort of adults with the entire clinical spectrum of TBE. We further found a trend of lower prevalence of homozygous wild genotype (p = 0.071) and wt allele (p = 0.096) in the adults with moderate and severe TBE. Therefore, it is tempting to speculate that TLR3 may play both beneficial and detrimental roles in pathogenesis of TBE: the carriers of wild type allele are more prone to develop clinical TBE; however, when the virus is already in the brain, TLR3 seems to play a protective role. This could also provide an explanation as to why no association between *TLR3* polymorphisms and clinical TBE was found in the cohort of adults with severe TBE when compared to the controls. It is striking that similar findings were observed in animal models with WNV [28], [29]. TLR3-deficient mice were more resistant to WNV after intraperitoneal inoculation but not after direct injection of WNV into the brain [28]. Our findings further support a benefit from the therapies directed at restoring the functional defect of TLR3.

TBE is associated with the infiltration of T cells and the macrophages (cell types known to express CCR5) into the brains of patients infected with TBEV [30]. The amount of the total protein in CSF is usually increased during TBE, and its elevation indirectly reflects the degree of the permeability of blood brain barrier [31]. Multivariate logistic regression model was employed to investigate the association of these laboratory parameters, demographic, and genetic findings with disease severity. Age, gender, and total protein in CSF were established as associated with severity of TBE, which is in line with other clinical studies [1], [5], [14], [31].

In animal models, other factors contributing to disease severity have been shown besides the host-dependent risk factors, such as the virulence of the particular strain, which could be inconsistent with the TBEV subtype [32], the inoculation dose [33], and the immunomodulatory compounds of tick saliva inoculated with virus into the skin [4]. Animal models are also commonly used to elucidate the mechanism of disease development following TBEV infection *in vivo* and suggest that CNS pathology during TBE is primarily driven by immune response and inflammatory reactions. A key role of CD8+ T cells in the immunopathology of TBE, as demonstrated by the prolonged survival of severe combined immunodeficiency (SCID) or CD8^−/−^ mice following infection compared with immunocompetent mice or mice with transferred CD8+ T cells has been shown by Ruzek et al. [34]. Another study found that TNF-α levels were significantly increased in the brains and in serum of mice that died following TBEV infection [35]. The most recent animal study showed that neutralizing antibody response is crucial for preventing fatality, while high expression of various cytokines/chemokines during TBE mediates immunopathology and is associated with a more severe course of the infection [36]. Furthermore, this study demonstrated that mice of the same age, gender, nutritious status, and TBEV inoculum, showed different TBE severity depending on genetic background, strongly supporting our findings.

A major limitation of our study is the lack of a TBEV seropositive asymptomatic cohort. Therefore, it remains unknown if *CCR5* and *TLR3* polymorphisms predispose to an increased susceptibility to TBEV infection or to the development of clinical illness. However, because TBEV seropositivity and mutations under consideration, especially *CCR5Δ32*, are both uncommon, a study including a sufficient number of asymptomatic TBEV seropositive individuals will need a large-scale multicentral approach.

In conclusion, this study confirms that polymorphisms in the *CCR5* and *TLR3* genes are risk factors for the development of clinical TBE. It also shows that the role of *TLR3* polymorphism differs in different age groups, and may be linked to disease severity. Further studies are needed to clarify if *CCR5* and *TLR3* polymorphisms play a role in susceptibility to TBEV infection and to further elucidate its influence on the severity of TBE.
